# Virologic outcomes of HAART with concurrent use of cytochrome P450 enzyme-inducing antiepileptics: a retrospective case control study

**DOI:** 10.1186/1742-6405-8-18

**Published:** 2011-05-16

**Authors:** Jason F Okulicz, Greg A Grandits, Jacqueline A French, Jomy M George, David M Simpson, Gretchen L Birbeck, Anuradha Ganesan, Amy C Weintrob, Nancy Crum-Cianflone, Tahaniyat Lalani, Michael L Landrum

**Affiliations:** 1 Infectious Disease Clinical Research Program, Uniformed Services University of the Health Sciences, Bethesda, MD, USA; 2 Infectious Disease Service, Brooke Army Medical Center, San Antonio TX, USA; 3 Division of Biostatistics, University of Minnesota, Minneapolis, MN, USA; 4 NYU Comprehensive Epilepsy Center, New York, NY, USA; 5 Department of Pharmacy Practice and Administration, Philadelphia College of Pharmacy, Philadelphia, PA, USA; 6 Department of Neurology, Mount Sinai School of Medicine, New York, NY, USA; 7 International Neurologic & Psychiatric Epidemiology Program, Michigan State University, East Lansing, MI, USA; 8 Division of Infectious Diseases, National Naval Medical Center, Bethesda, MD, USA; 9 Infectious Disease Service, Walter Reed Army Medical Center, Washington, DC, USA; 10 Infectious Disease Clinic, Naval Medical Center San Diego, San Diego, CA, USA

## Abstract

**Background:**

To evaluate the efficacy of highly-active antiretroviral therapy (HAART) in individuals taking cytochrome P450 enzyme-inducing antiepileptics (EI-EADs), we evaluated the virologic response to HAART with or without concurrent antiepileptic use.

**Methods:**

Participants in the US Military HIV Natural History Study were included if taking HAART for ≥6 months with concurrent use of EI-AEDs phenytoin, carbamazepine, or phenobarbital for ≥28 days. Virologic outcomes were compared to HAART-treated participants taking AEDs that are not CYP450 enzyme-inducing (NEI-AED group) as well as to a matched group of individuals not taking AEDs (non-AED group). For participants with multiple HAART regimens with AED overlap, the first 3 overlaps were studied.

**Results:**

EI-AED participants (n = 19) had greater virologic failure (62.5%) compared to NEI-AED participants (n = 85; 26.7%) for the first HAART/AED overlap period (OR 4.58 [1.47-14.25]; P = 0.009). Analysis of multiple overlap periods yielded consistent results (OR 4.29 [1.51-12.21]; P = 0.006). Virologic failure was also greater in the EI-AED versus NEI-AED group with multiple HAART/AED overlaps when adjusted for both year of and viral load at HAART initiation (OR 4.19 [1.54-11.44]; P = 0.005). Compared to the non-AED group (n = 190), EI-AED participants had greater virologic failure (62.5% vs. 42.5%; P = 0.134), however this result was only significant when adjusted for viral load at HAART initiation (OR 4.30 [1.02-18.07]; P = 0.046).

**Conclusions:**

Consistent with data from pharmacokinetic studies demonstrating that EI-AED use may result in subtherapeutic levels of HAART, EI-AED use is associated with greater risk of virologic failure compared to NEI-AEDs when co-administered with HAART. Concurrent use of EI-AEDs and HAART should be avoided when possible.

## Background

Seizure disorders are common in HIV-infected individuals, with an incidence of up to 11% in several cohort studies[1-3]. Antiepileptic drugs (AEDs) are frequently prescribed to patients with HIV not only for pre-existing epilepsy, but also in the setting of CNS opportunistic infections and for other neurologic and psychiatric conditions including neuropathy, refractory headaches, depression, and bipolar disorder[4]. Concurrent use of highly-active antiretroviral therapy (HAART) and AEDs has the potential for highly complex and clinically significant drug interactions.

Several first-generation AEDs, including phenytoin, carbamazepine, and phenobarbital are metabolized by the cytochrome P450 (CYP450) enzyme system. This pathway of drug metabolism is also utilized by protease inhibitors (PIs), non-nucleoside reverse transcriptase inhibitors (NNRTIs), and the CCR5 inhibitor maraviroc[4,5]. In addition to competing for enzyme binding sites, some antiretrovirals and AEDs intrinsically induce CYP450 metabolism with the potential to decrease blood levels of both agents. As a consequence, this drug interaction may limit the effectiveness of HAART and predispose patients to adverse HIV treatment outcomes, including virologic failure, accumulation of antiretroviral drug resistance mutations, and HIV disease progression. In turn, lower AED blood levels may also have deleterious effects due to lack of efficacy, including loss of seizure control or inadequate control of neuropathic pain.

In many parts of the world where HIV is highly prevalent, such as sub-Saharan Africa and parts of Asia, only CYP450 enzyme-inducing AEDs (EI-AEDs) are available[6]. Thus, treating both HIV and epilepsy in these areas may lead to suboptimal treatment of both conditions due to the potential for clinically significant drug interactions. The US Department of Health and Human Services (DHHS) guidelines[7] recommend considering alternative anticonvulsants or monitoring drug levels, while WHO guidelines[8] recommend cautious use, drug level monitoring, or avoidance of these combinations. Since there are inadequate clinical data and the virologic efficacy is largely unknown for HAART in the setting of concurrent EI-AED use, we performed a retrospective case control study to determine the virologic outcomes in participants with concomitant HAART/EI-AED use in the US military HIV Natural History Study (NHS).

## Methods

Participants were identified in the database of over 5,000 patients enrolled in the NHS since 1986, a cohort of consenting military members, retirees, and beneficiaries 18 years or older with HIV[9,10]. Individuals are seen approximately every 6 months at participating United States military treatment facilities. Data are systematically collected, including demographic characteristics, information on medication use, laboratory data, and reports of clinical events with medical record confirmation.

The NHS database was searched for individuals on concurrent HAART and EI-AEDs, which included phenytoin, carbamazepine, and phenobarbital. HAART regimens were PI- or NNRTI-based as previously defined[10]. Regimens consisting only of triple nucleoside reverse transcriptase inhibitors (NRTIs) were excluded due to the lack of drug interactions between EI-AEDs and the NRTI class. Participants included were those taking a PI- or NNRTI-based HAART regimen for ≥6 consecutive months and during this period were taking an EI-AED drug for ≥28 consecutive days. Since participants in the EI-AED group may have taken medications that can affect CYP450 metabolism other than HAART and EI-AEDs, use of rifamycin antibiotics (CYP450 inducers) and azole antifungals (CYP450 inhibitors) were reported.

### Comparison Groups

Two control groups were used for comparisons to the EI-AED group, the first consisted of participants prescribed AEDs that are not CYP450 enzyme-inducing, the NEI-AED group. NEI-AEDs included levetiracetam, lamotrigine, zonisamide, ethosuxamide, topiramate, gabapentin, tiagabine, and pregabalin. Oxcarbamazepine (a weak CYP450 inducer) and valproic acid (a CYP450 inhibitor) were excluded. Since participants in the EI-AED group were prescribed AEDs primarily for seizure treatment and prophylaxis as well as neuropathy, participants in the NEI-AED group were restricted to those taking these drugs for the same indications. Analogous to the EI-AED group, the NEI-AED group was required to have a HAART duration ≥ 6 months and an AED overlap during the HAART period of ≥28 days.

The second control group was a subgroup of all participants on HAART without any AED use in the NHS (non-AED group), matched (10:1) to each EI-AED patient according to year of HAART initiation and number of previous HAART regimens. Participants in this control group must have been on their HAART regimen for ≥ 6 months to be included. Since all cases in the EI-AED group had a documented date of HIV infection prior to the year 2000, all potential controls were limited to those with dates of HIV infection prior to 2000.

### Virologic Outcomes

Virologic failure was defined as having all plasma viral loads (VLs) in the first 6 months of HAART (minimum of 2 values) ≥400 copies/mL and/or the participant having 2 consecutive VLs ≥400 copies/mL after 6 months of HAART. Other virologic outcomes assessed included the percentage of participants with VL <400 copies/mL at 6 and 12 months of HAART, and the average of VL (log_10_) for each individual within the HAART period. For virologic suppression at 6 and 12 months, the VL closest to 6 and 12 months after HAART initiation, respectively, were used.

### Statistical Methods

Logistic regression was used to compare the proportion with virologic failure and viral suppression after starting HAART in the EI-AED and each of the comparison groups. For each outcome, the number and percent of participants with the outcome are reported with the relative odds and 95% CI, cases versus controls. Analysis of variance and covariance was used to compare cases and controls for average VLs during HAART. Mean case-control differences are reported ± standard error (SE). Because some individuals in the EI-AED and NEI-AED groups had multiple HAART episodes with concurrent AED use, GEE analyses was used to compare virologic outcomes using multiple HAART/AED overlap episodes (up to the first 3 episodes). For binary outcomes, a logic link was used in the GEE analyses; for continuous outcomes a normal link was used.

Both univariate and multivariate analyses were performed. For the analyses comparing the EI-AED and NEI-AED groups, a model adjusting for year of and VL prior to HAART initiation was performed. For comparison of the EI-AED and non-AED groups, adjustment was made only for prior VL, since cases were matched to controls for year starting HAART. SAS, version 9.2 was used for all analyses; PROC GENMOD was used for the GEE analyses.

## Results

### Baseline and Demographic Factors

Based on inclusion criteria, 19 participants were treated concurrently with EI-AEDs and HAART, with 12, 6, and 1 taking phenytoin, carbamazepine, and phenobarbital for the first HAART/EI-AED overlap period, respectively. EI-AEDs were used for seizure disorder in 17 of 19 participants; further characterization of seizure disorders included CNS toxoplasmosis (2 with seizures, 1 for prophylaxis), herpes simplex meningitis/encephalitis (n = 2), progressive multifocal leukoencephalopathy (n = 1), seizures following a motor vehicle accident (n = 1), and the remainder with seizure disorders without further characterization (n = 10). Two individuals used EI-AEDs for neuropathic pain. For the NEI-AED group, 85 participants met inclusion criteria; 82 received gabapentin, 2 pregabalin, and 1 levetiracetam at the first overlap. The majority of participants were prescribed NEI-AEDs for the indication of neuropathic pain (81/85, 95%), with the remainder for seizure disorder (4/85, 5%). Two participants were prescribed rifampin and 1 participant had a history of itraconazole use, however none of these medications were prescribed during the HAART periods investigated in this study.

Demographic factors including age at HIV diagnosis and race were similar between the EI-AED, NEI-AED and non-AED groups. However, participants in the EI-AED group were younger at first HAART/AED overlap compared to the NEI-AED group (40.1 vs. 45.1 years, P = 0.027; Table 1), reflective of the EI-AED group starting HAART during an earlier calendar year (median 1998 versus 2003). Mean CD4 cell count and log_10 _VL at HAART initiation were similar between the 3 groups, although VL levels tended to be higher in the EI-AED compared to the NEI-AED group (3.8 versus 3.1 log_10 _copies/mL; P = 0.075). The EI-AED group had a higher proportion of individuals with AIDS-defining events prior to the HAART period analyzed, however this was only significant in comparison to the non-AED group (57.9% vs. 21.1%; P < 0.001). The majority of participants in all groups were HAART treatment experienced, however the EI-AED group had a higher percentage of HAART-naive individuals (21.1%) compared to the NEI-AED group (7.1%; P = 0.01).

**Table 1:** Characteristics of HIV Subgroups

Characteristic	EI-AED	NEI-AED	Non-AED	EI-AED vs. NEI-AED (p-values)	EI-AED vs. Non-AED (p-values)
**Number, n**	19	85	190		
**Demographics (n, % or mean ± SD)**					
Age at HIV diagnosis (y)	30.2 ± 10.0	32.5 ± 8.9	30.0 ± 7.9	0.331	0.912
Age at first HAART/AED overlap or index HAART (y)	40.1 ± 8.7	45.1 ± 9.0	38.7 ± 9.1	0.027	0.522
Female	1 (5.3)	13 (15.3)	18 (9.5)	0.249	0.544
Race/ethnicity				0.323	0.137
European American	13 (68.4)	46 (54.1)	89 (46.8)		
African American	6 (31.6)	32 (37.6)	84 (44.2)		
Other	0 (0.0)	7 (8.2)	17 (8.9)		
Year of HIV diagnosis (median, range)	1988 (1985 - 2000)	1990 (1986 - 1999)	1990 (1985 - 2000)	0.145	0.122
CD4+ at HIV diagnosis (cells/uL)	632 ± 398	525 ± 321	483 ± 235	0.340	0.068
CD4+ at first HAART/AED overlap or index HAART (cells/uL)	310 ± 290	380 ± 247	364 ± 232	0.329	0.392
Viral load (log10) at first HAART/AED overlap or index HAART (copies/mL)	3.8 ± 1.6	3.1 ± 1.4	3.6 ± 1.4	0.075	0.572
AIDS-defining event prior to first HAART/AED overlap or index HAART	11 (57.9)	35 (41.2)	40 (21.1)	0.187	<.001
**HAART regimen at first HAART/AED overlap or index HAART (n, %)**				0.189	0.543
PI-based	12 (63.2)	35 (41.2)	117 (61.6)		
NNRTI-based	5 (26.3)	29 (34.1)	36 (18.9)		
PI + NNRTI	2 (10.5)	21 (24.7)	37 (19.5)		
**Number of HAART/AED overlap periods (n, %)**				0.343	---
1	7 (36.8)	47 (55.3)	---		
2	5 (26.3)	17 (20.0)	---		
≥ 3	7 (36.8)	21 (24.7)	---		
**Months of HAART/AED overlap (median, range)**					
First overlap period	7.0 (1.0 - 96.4)	9.1 (1.3 - 65.4)	---	0.231	---
All overlap periods	21.3 (1.0 - 155.4)	22.1 (1.6 - 120.3)	---	0.798	---
**Year of first HAART/AED overlap or index HAART (median, range)**	1998 (1996 - 2006)	2003 (1996 - 2009)	1998 (1996 - 2006)	0.002	1.000
**HAART use prior to first HAART/AED overlap or index HAART (n, %)**				0.010	0.523
No prior HAART	4 (21.1)	6 (7.1)	40 (21.1)		
<1 year	4 (21.1)	7 (8.2)	37 (19.5)		
1-2 years	2 (10.5)	7 (8.2)	31 (16.3)		
2-3 years	4 (21.1)	6 (7.1)	17 (8.9)		
>3 years	5 (26.3)	59 (69.4)	65 (34.2)		
**Duration of first HAART/AED overlap or index HAART (n, %)**				0.144	0.495
<1 year	10 (52.6)	22 (25.9)	69 (36.3)		
1-2 years	4 (21.1)	22 (25.9)	63 (33.2)		
2-3 years	2 (10.5)	15 (17.6)	31 (16.3)		
>3 years	3 (15.8)	26 (30.6)	27 (14.2)		

EI-AED, enzyme-inducing antiepileptics (phenytoin, carbamazepine, phenobarbital); NEI-AED, antiepileptics that are not enzyme-inducing; Non-AED, subgroup of all subjects in the cohort (exclusive of other groups) matched according to year of HAART start and number of previous HAART regimens; PI, protease inhibitor; NNRTI, non-nucleoside reverse transcriptase inhibitor

In evaluating HAART/AED overlap periods, 7 (36.8%) EI-AED participants had a single period of overlap, while 5 (26.3%) and 7 individuals (36.8%) had 2 or ≥3 overlap periods, respectively. The number of overlaps was similar for the NEI-AED group. The duration of first HAART/AED overlap was no different between the groups, with 7.0 months (range 1.0-96.4) overlap in the EI-AED group compared to 9.1 months (1.3-65.4) for the NEI-AED group (P = 0.231). The groups were also similar when all eligible HAART/AED periods were considered, with 21.3 (1.0-155.4) and 22.1 (1.6-120.3) months of overlap for the EI-AED and NEI-AED groups, respectively (P = 0.798).

### EI-AED Group versus NEI-AED Group

In comparing outcomes for the first HAART/AED overlap, virologic failure was significantly greater in the EI-AED group (62.5%) compared to the NEI-AED group (26.7%; P = 0.009; Table 2). The average log_10 _VL during the overlap period was also higher in the EI-AED group (3.3 ± 1.3 vs. 2.4 ± 1.2; P = 0.006). The percentage of participants with VL <400 copies/mL was significantly lower in the EI-AED group compared to the NEI-AED group at 6 months (33.3% vs. 71.4%; P = 0.016) and 12 months (36.4% vs. 75%; P = 0.018), respectively.

**Table 2:** Virologic Outcomes of EI-AED Compared to NEI-AED Subjects

	EI-AED (N = 19)	NEI-AED (N = 85)	OR (95% CI) or Difference ± SE	Adjusted for Year of and VL at HAART Initiation OR (95% CI) or Difference ± SE
**First HAART/AED Overlap**				
Virologic failure	10/16 (62.5%)	20/75 (26.7%)	4.58 (1.47 - 14.25) P = 0.009	4.67 (0.92 - 23.62) P = 0.062
Average VL during period (log10)	3.3 ± 1.3 (n = 19)	2.4 ± 1.2 (n = 84)	0.8 ± 0.3; P = 0.006	0.2 ± 0.3; P = 0.376
VL <400 at 6 months	4/12 (33.3%)	50/70 (71.4%)	0.20 (0.05 - 0.74) P = 0.016	0.35 (0.06 - 2.07) P = 0.247
VL <400 at 12 months	4/11 (36.4%)	42/56 (75.0%)	0.19 (0.05 - 0.75) P = 0.018	0.17 (0.02 - 1.42) p = 0.102
All HAART/AED Overlaps*				
Virologic failure	19/30 (63.3%)	34/122 (27.9%)	4.29 (1.51 - 12.21) P = 0.006	4.19 (1.54 - 11.44) P = 0.005
Average VL during period (log10)	3.3 ± 1.3 (n = 34)	2.5 ± 1.3 (n = 142)	0.9 ± 0.3 P = 0.005	0.7 ± 0.3 P = 0.007
VL <400 at 6 months	8/28 (28.6%)	84/121 (69.4%)	0.17 (0.06 - 0.53) P = 0.002	0.25 (0.07 - 0.86) P = 0.028
VL <400 at 12 months	9/23 (39.1%)	71/96 (74.0%)	0.21 (0.07 - 0.61) P = 0.004	0.22 (0.06 - 0.75) P = 0.016

OR (odds ratio): Odds of virologic event for EI-AED cases versus odds for NEI-AED controls; VL, viral load (copies/mL); * Up to three intervals used per subject

Results were similar when multiple HAART/AED overlap periods per individual were included in the analyses, which added approximately twice the number of HAART/AED episodes (Table 2). Virologic failure was more common in HAART episodes for EI-AED (63.3%) compared to NEI-AED individuals (27.9%; P = 0.006) and the average log_10 _VL during the overlap period was significantly higher in the EI-AED group (3.3 ± 1.3 vs. 2.5 ± 1.3; P = 0.005). The percentage of participant HAART episodes with VL < 400 copies/mL was also lower in the EI-AED group compared to the NEI-AED group at 6 months (28.6% vs. 69.4%; P = 0.002) and 12 months (39.1% vs. 74%; P = 0.004).

### EI-AED Group versus NEI-AED Group - Multivariate Analyses

Analysis of virologic outcomes adjusting for year of and VL at HAART initiation yielded similar odds ratios to that in the univariate analyses. This was true for both analyses of the initial overlap period and in using multiple overlaps. However, only for the multiple overlap analyses were the odds ratios significantly different from one. The estimated odds ratio for virologic failure for the EI-AED group compared to the NEI-AED group using multiple episodes was 4.19 (95% CI [1.54-11.44]; P = 0.005).

### EI-AED Group versus non-AED Group - Univariate and Multivariate Analyses

For the EI-AED group, virologic failure was higher compared to the non-AED group (62.5% vs. 42.5%) in univariate analysis, but the result did not reach statistical significance (P = 0.134; Table 3). Similar results were observed for other virologic outcomes, with less virologic suppression to <400 copies/mL at 6 months (OR 0.40 [0.12-1.38]; P = 0.146) and 12 months (OR 0.32 [0.09-1.16]; P = 0.083) for the EI-AED group compared to the non-AED group, respectively. Correspondingly, average log_10 _VL during the overlap period was higher in the EI-AED group than in the non-AED group (3.3 ± 1.3 vs. 2.9 ± 1.2; P = 0.198). When adjusted for VL at HAART initiation, there was significantly greater virologic failure in the EI-AED group compared to the non-AED group (OR 4.30 [1.02-18.07]; P = 0.046). The adjusted odds of virologic suppression was lower in the EI-AED than in the non-AED group at 6 and 12 months, with the latter being statistically significant (OR 0.17 [0.03-0.89]; P = 0.036).

**Table 3:** Virologic Outcomes of EI-AED Compared to Non-AED Subjects

	EI-AED (N = 19)	Non-AED (N = 190)	OR (95% CI) or Difference ± SE	Adjusted for VL at HAART Initiation OR (95% CI) or Difference ± SE
**First HAART/AED Overlap**				
Virologic failure	10/16 (62.5%)	62/146 (42.5%)	2.26 (0.78 - 6.54) P = 0.134	4.30 (1.02 - 18.07) P = 0.046
Average VL during period (log10)	3.3 ± 1.3 (n = 19)	2.9 ± 1.2 (n = 184)	0.4 ± 0.3; P = 0.198	0.3 ± 0.3; P = 0.195
VL <400 at 6 months	4/12 (33.3%)	89/160 (55.6%)	0.40 (0.12 - 1.38) P = 0.146	0.30 (0.06 - 1.48) P = 0.139
VL <400 at 12 months	4/11 (36.4%)	80/125 (64.0%)	0.32 (0.09 - 1.16) P = 0.083	0.17 (0.03 - 0.89) P = 0.036

OR (odds ratio): Odds of virologic event for EI-AED cases versus odds for non-AED controls; VL, viral load (copies/mL)

## Discussion

HIV-infected patients commonly require treatment with AEDs due to neurologic and psychiatric conditions. Drug interactions between EI-AEDs and HAART are highly complex and may result in loss of efficacy for one or both treatments. In examining this interaction retrospectively in a military HIV cohort with free access to healthcare and medications, we found greater virologic failure in individuals taking EI-AEDs compared to NEI-AEDs when used in combination with HAART. Since first line agents for epilepsy in most low and middle income countries are limited to EI-AEDs, the clinical ramifications of HAART/EI-AED drug interactions may be substantial.

Despite the widespread use of EI-AEDs and the potential for significant drug interactions with HAART, clinical studies are extremely limited[11]. A randomized, parallel-arm study examined the pharmacokinetic interaction between lopinavir/ritonavir (400 mg/100 mg twice daily) and phenytoin (300 mg daily) in healthy volunteers[12]. In the first arm of 12 participants, the addition of phenytoin reduced the area under the concentration-time curve (AUC) of lopinavir and ritonavir by 33% and 28%, respectively after 12 days of overlap compared to the pre-phenytoin period. Notably, the effect of increased lopinavir clearance secondary to CPY3A4 induction by phenytoin was not offset by the presence of low dose ritonavir used as a "boosting" agent. The second arm of 8 participants showed a 31% reduction in phenytoin AUC after the addition of lopinavir/ritonavir demonstrating a two-way drug interaction between classes. A similar result was shown in a randomized, crossover study of 18 healthy individuals receiving either efavirenz (600 mg daily) or carbamazepine (titrated to 400 mg daily) followed by 14-21 days of overlap with the other drug[13]. Compared to pre-overlap levels, efavirenz AUC and minimum (Cmin) and maximum (Cmax) concentrations were reduced by approximately 17% to 43% while carbamazepine AUC decreased by 27%. Though the majority of drug-drug interactions result in reduced plasma concentrations, carbamazepine toxicity may occur secondary to inhibition of CYP3A4 when used with low dose ritonavir[4,14]. Since most studies were performed in healthy volunteers, extrapolation of these findings to patients with HIV infection and epilepsy is difficult because the clinical implications of these interactions have not been adequately studied.

This is the first study demonstrating clinically meaningful outcomes in participants receiving overlapping treatment with EI-AEDs and HAART. The impact is so robust, that we were able to demonstrate this despite the small number of individuals receiving EI-AEDs. Since it is more difficult to enter military service with pre-existing epilepsy, the overall incidence of epilepsy is low in our cohort. Yet, the close follow-up in this prospective observational cohort makes it uniquely ideal for an assessment of clinical consequences of this interaction. Despite the small number of participants taking EI-AEDs in our study, these agents are still commonly used even in the United States. EI-AEDs are favored by some insurance plans due to their lower cost, so it is likely that a cohort with a higher prevalence of epilepsy would have included more participants on EI-AEDs. It is notable that of the 21 participants diagnosed with a seizure disorder in this study, 17 were taking EI-AEDs.

The comparison of EI-AEDs versus NEI-AEDs combined with HAART in our study showed worse virologic outcomes in the EI-AED group. The inclusion criteria for the NEI-AED group were chosen to best approximate the participants in the EI-AED group, specifically targeting use of NEI-AEDs for the indications of seizure disorder or neuropathic pain. In cases where the specific indication for AED use was known, the majority of individuals were prescribed AEDs in the setting of CNS opportunistic infections. The relatively small number of individuals in the EI-AED group limited the power of the study. This was likely due to the increased availability of newer AEDs that are not CYP450-enzyme inducing over the past decade. Other limitations include the differing proportions of seizure disorders and neuropathic pain in the two groups. As a reflection of this, the drugs in the NEI-AED group are agents commonly used for neuropathic pain. Multivariate analyses were performed in an attempt to minimize some of the differences in HAART period between groups, with results demonstrating worse virologic outcomes in the EI-AED group. Other unmeasured factors included HIV drug resistance, potency of HAART regimens, adherence to both drug classes, absence of ARV and AED blood levels, and the inability to study individual EI-AED and ARV pairings due to small sample size. The EI-AED group also had a higher percentage of AIDS events and higher VL prior to the first AED/HAART overlap compared to NEI-AED group. This suggests that the EI-AED group may have more advanced HIV disease and greater risk of virologic failure. It is important to note, however, that the EI-AED group had less treatment experience than the NEI-AED group, with 42% and 15% having <1 year of HAART experience, respectively. This difference in treatment experience in the EI-AED group may potentially offset the risk of treatment failure posed by having a higher percentage of prior AIDS events.

For comparison with both NEI-AED and non-AED control groups, the EI-AED group had consistently worse virologic outcomes, especially in multivariate analyses. Non-AED individuals fared better than EI-AED participants, however the results were significant only after adjustment for VL at HAART initiation. There are several unmeasured factors that may have contributed to these findings including medication doses and adherence, HIV drug resistance, and other uncharacterized variables unique to patients with seizure disorders or neuropathic pain. Measures of AED efficacy, including seizure control, were not completely captured. Our initial hypothesis was that concurrent HAART/EI-AED use would lead to subtherapeutic blood levels of HAART, elevated VLs, and eventually virologic failure. We chose a minimum HAART/AED overlap period of ≥28 days due to the small number of patients exposed to EI-AEDs for any duration. The median duration for all overlaps was 9 months for the EI-AED group. Since epilepsy and seizure disorders typically require long-term, if not life-long treatment, many patients will be taking EI-AEDs and HAART for extended periods of time. Even though the percentage of participants with virologic failure was high at 63.3%, it is possible that the HAART/EI-AED overlap time was insufficient to develop regimen failure for some individuals and additional failures would occur with continued use of both classes.

The introduction of EI-AEDs in patients with HIV may complicate a regimen that is already subject to other challenges from drug interactions. For example, the burden of tuberculosis in sub-Saharan Africa requires many HIV-infected patients to receive concurrent antituberculous treatment (ATT). The cornerstone of ATT regimens is the rifamycin class of antibiotics. As inducers of CYP450, rifamycins can also enhance the metabolism of AEDs and antiretrovirals, adding further management challenges[15]. Compared to rifampin, rifabutin has less CYP450 induction and is favored for ATT in the setting of HAART. In the current study, no participants were treated for active tuberculosis during the study period.

The World Health Organization's list of essential medicines includes the EI-AEDs carbamazepine, phenytoin, and phenobarbital[16]. In addition to the availability of only EI-AEDs in many areas of the world, the expanding use of AEDs in patients with HIV has made the management of HIV and comorbid conditions challenging in these locations, as well as in more developed countries. For example, distal sensory polyneuropathy (DSP), often treated with AEDs, occurs in up to 57% of patients with HIV and the risk of developing DSP is increased with underlying nutritional deficiencies[17-19].

In the setting of concurrent HAART/EI-AED use, the US Department of Health and Human Services (DHHS) guidelines[7] recommend providers consider use of alternative agents and/or monitoring of blood levels of HAART/EI-AEDs. Therapeutic drug monitoring (TDM) of HAART is not routinely recommended for the management of HIV patients. However, DHHS guidelines suggest that TDM may be useful in situations with clinically significant drug-drug interactions that may result in reduced efficacy, including use of certain AEDs. TDM may identify reduced blood levels of HAART as a result of drug-drug interactions prompting the provider to consider increasing the dose of ARVs. However, this may lead to a higher rate of adverse effects and ultimately impact drug tolerance and adherence.

Despite the potential merits of this approach, TDM has several limitations including cost and limited availability. According to a recent Cochrane review[20], TDM trials are generally small and underpowered, have short follow-up time, and poor compliance with TDM recommendations. TDM trials have also been performed in countries with higher income and may not be generalized to resource-limited settings. Since the majority of EI-AED use is in low and middle income countries due to the greater cost of NEI-AEDs, TDM is unlikely to be an option for clinicians in these areas.

In settings where EI-AEDs must be used, it is important to recognize treatment failure early with frequent VL monitoring and clinical assessments for efficacy of HAART and EI-AEDs. HAART regimens composed of the integrase inhibitor raltegravir in combination with 2 NRTIs would enable clinicians to avoid drug-drug interactions, however raltegravir may not be available in many areas. Although there are concerns that a triple NRTI regimen may be less durable compared to other HAART regimens, this may be another reasonable option given the lack of drug interactions between NRTIs and EI-AEDs[21].

For the treatment of epilepsy in the setting of HIV infection, alternative agents to EI-AEDs should be administered if available. Valproic acid is available in many regions, however this drug is a weak inhibitor of CYP450 and may lead to increased HAART levels and toxicity, especially when used with lopinavir/ritonavir[4,22]. Due to its additional property as a non-selective histone deacyltase (HDAC) inhibitor, *in vitro *studies indicate valproic acid may increase HIV outgrowth from resting CD4+ T cells[23]. However, the *in vivo *effects and clinical relevance have not been firmly established[24,25]. Despite the potential concerns, valproic acid appears to be a safe alternative to EI-AEDs when used with HAART[26].

## Conclusions

EI-AEDs should be avoided in favor of NEI-AEDs in patients requiring concurrent HAART and AED therapy due to the higher potential of virologic failure and reduced efficacy. In areas where EI-AED use cannot be avoided, closer and more frequent monitoring of HIV and seizure control is warranted. Alternative HAART regimens, such as triple NRTIs or integrase-based regimens, and use of TDM may be beneficial in managing or avoiding these complex drug interactions when available. In low to middle income regions such as sub-Saharan Africa and parts of Asia, treatment of HIV and comorbid epilepsy and other neurologic conditions will continue to pose great challenges until additional resources become available, such as NEI-AEDs and a wider repertoire of antiretrovirals.

## Competing interests

JFO, GAG, DMS, GLB, AG, ACW, NC, TL, and MLL declare that they have no competing interests.

JAF has served on the scientific advisory board of UCB, Johnson & Johnson, Eisai, Novartis, Valeant, Icagen, Intranasal, Sepracor, and Marinus. Dr. French is the president of the Epilepsy Study Consortium that receives funding from multiple pharmaceutical companies.

JMG is a consultant for Pfizer.

## Authors' contributions

All authors participated in the design of the study and manuscript preparation. GAG performed the statistical analysis. All authors read and approved the final manuscript.
